# Deformities due to Leprosy in Children under Fifteen Years Old as an Indicator of Quality of the Leprosy Control Programme in Brazilian Municipalities

**DOI:** 10.1155/2013/812793

**Published:** 2013-03-19

**Authors:** Francisco Carlos Félix Lana, Angélica da Conceição Oliveira Coelho Fabri, Fabiana Nascimento Lopes, Ana Paula Mendes Carvalho, Fernanda Moura Lanza

**Affiliations:** ^1^Nursing School, Federal University of Minas Gerais, Avenida Alfredo Balena, 190/402, Santa Efigênia, 30130-100 Belo Horizonte, MG, Brazil; ^2^Nursing School, Federal University of São João DelRei, Rua Sebastião Gonçalves Coelho, 400, Chanadour, 35501-293 Divinópolis, MG, Brazil

## Abstract

The present study aims at analysing the degree of deformity in leprosy cases diagnosed in children under 15 years old and its relationship with operational and epidemiological factors. This epidemiological cross-sectional study was carried out at municipalities of three microregions in a Brazilian hyperendemic area. Data between 1998 and 2010 was collected from the Information System for Notifiable Diseases database. The average coefficient of detection was 32.96/100.000 inhabitants; 7.61% of new cases were diagnosed in children under 15 years old; 5% in this age group were grade 2 deformity at diagnosis. Prevalence of leprosy cases in children under 15 years old with deformity was higher in males (PR = 2.65;*P* = 0.032; CI 95%: 1.09–6.45) and in multibacillary patients (PR = 14.68;*P* < 0.001; CI 95%: 3.54–60.87) and lower when the detection mode was passive (PR = 0.73,*P* = 0.47, CI 95%: 0.31–1.73). Such context suggests high transmissibility and early exposure to *Mycobacterium leprae* since a lot of cases were diagnosed in children under fifteen years old and the incubation period of the leprosy bacillus varies from 02 to 07 years. This situation contributes to maintaining the chain of disease transmission in the area and indicates that health care services should intensify leprosy control.

## 1. Introduction

Notwithstanding the reduction in the number of leprosy cases, the disease remains an important public health issue in many countries. In the Americas, Brazil is still the most endemic country. In 2010 Brazil's coefficient of detection was of 18.29/100.000 inhabitants in the general population and of 1.29/100.000 in the population under 15 years old. Among the new cases of the disease, 6.4% showed grade 2 deformity [1].

Leprosy Control Programs are constantly changing. The current World Health Organization (WHO) strategy is to reduce the number of cases among those under 15 years old and to achieve a 35% reduction in the new cases detection rate with grade 2 deformity by the end of 2015, 2010 being the baseline. These indicators help to measure quality of care and to monitor the Leprosy Eradication Program [2]. Nevertheless, according to recent studies, Brazil will not meet the WHO leprosy elimination target of reducing the new cases detection rate with grade 2 deformity by 2015 [3].

Leprosy detection in children under 15 years old is a strong indicator of recent transmission by active sources of infection [4] and suggests that the population is being exposed to cases not yet diagnosed by the health services. Conditions of high transmissibility and early exposure to *Mycobacterium leprae* increase the chances of developing the disease [5].

Early and prolonged exposure to untreated leprosy cases and late diagnosis might favour the occurrence of deformities. The occurrence of deformities can be applied to indirectly measure the magnitude of leprosy, since most patients do not develop deformity in the early stages of the disease [6].

Late diagnosis and active transmission of leprosy are factors that interfere with effective control and elimination of the disease [7]. In Brazil, the health reform, the creation of the Unified Health System (in Portuguese, SUS), and the implementation of a Family Health Strategy (in Portuguese, ESF) increased the access to leprosy diagnosis [8]. It is known that the operational capacity of the health services influences the achievement of early diagnosis [9].

The Brazilian strategies for leprosy control include integration of leprosy control actions in the primary health care [10], early diagnosis, timely treatment of diagnosed cases, and surveillance of household contacts [11]. In addition, the identification of clusters (areas at a higher risk of developing leprosy) enables health professionals to focus on a geographically continuous area, ensuring more effective epidemiological control. Cluster number 6 encompasses municipalities in the state of Minas Gerais, some of them in the Jequitinhonha Valley [12].

The present study aims at analysing the degree of physical deformity in leprosy cases diagnosed in children under 15 years old and its relation to operational and epidemiological factors.

## 2. Methodology

This is a cross-sectional study carried out at the Almenara, Araçuaí, and Diamantina microregions located in the Jequitinhonha Valley, northeast of the State of Minas Gerais. Almenara and Araçuaí were selected because of rates of new leprosy cases and are considered a priority in the state's disease control; Diamantina presented a high percentage of leprosy cases with deformity. The three microregions comprise 16, 6, and 15 municipalities, respectively.

Data collected relates to cases of leprosy diagnosed among the general population and in children under 15 years old from 1998 to 2010. Data was obtained via the Information System for Notifiable Diseases (in Portuguese, SINAN) and the Clinical Dermatology Coordination (in Portuguese, CEDS) from the Minas Gerais Department of Health (in Portuguese, SES/MG). Information regarding regions' resident population was obtained from the Brazilian Institute of Geography and Statistics (in Portuguese, IBGE).

A 14-year-period study was chosen to minimize possible variations in epidemiological indicators related to inconsistencies in the SINAN database, such as those mentioned in the study to evaluate the leprosy surveillance system in Brazil [13] and the operational capacity of the health services.

Epidemiological profiles were analysed through indicators established by the World Health Organization, such as the absolute number and the proportion of cases in patients under 15 years old, the absolute number and the proportion of new cases with grade 2 deformity, the absolute number and the proportion of female cases, and the absolute number and the proportion of multibacillary patients among new cases.

The following epidemiological and operational indicators established by the Brazilian Department of Health such as [14] proportion of cured leprosy cases with grade 2 deformity, proportion of new leprosy cases with deformity grade assessed at diagnosis, and proportion of cured cases during the year of assessment were also used.

Treatment and analysis of data were carried out by software *Statistical Package for Social Sciences *(SPSS) version 18.0 and *Statistical Software for Professionals* (STATA), version 11.0.

The degree of deformity at diagnosis was the study's dependent variable. It is determined by the evaluation of eyes, hands, and feet and varies on a scale from 0 to 2 where 0 means no deformity found, 1 means loss of sensation in hands or feet, and 2 means lesion or visible deformity [2].

Gender, operational classification, clinical type, skin smear test, and detection mode were the independent variables.

Factors associated with the degree of deformity at diagnosis were analysed via the calculation of prevalence ratio (PR) using Poisson regression with robust error variance since the study design is transversal and the analysed outcome is common [15, 16].

*P*  value < 0.20 in the bivariate analysis and epidemiological and biological plausibility were the criteria for including variables in the multivariate model. After selection the variables were entered one at a time in the regression model and the ones that lost their significance were excluded. Statistical significance level considered was 5% (*P* < 0.05).

The National Health Council guidelines and standards for research with human beings according to Resolution 196/96 were observed. The research projects related to this study were approved by the Ethics in Research Committee of the Federal University of Minas Gerais (in Portuguese, COEP-UFMG) report nos. 149/07, ETIC 158/09, and ETIC 0512.0.203.000-10.

## 3. Results

**Figure 1:**
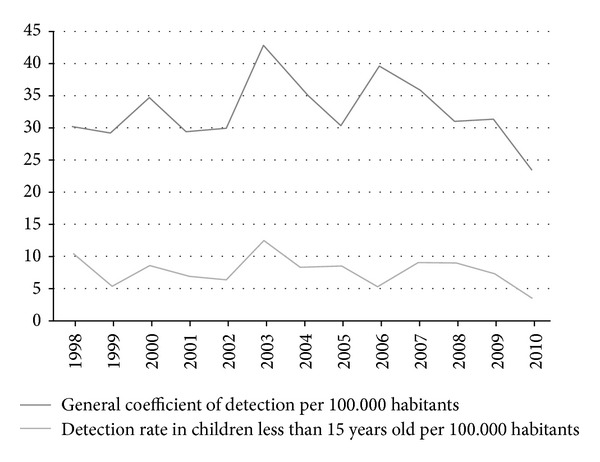
Coefficient of leprosy detection among the general population and in children under 15 years old per 100.000 in habitants in Almenara, Araçuaí, and Diamantina microregions between 1998 and 2010.

Between 1998 and 2010, 1838 leprosy cases in Almenara, Araçuaí, and Diamantina were reported to the SINAN. This corresponds to a mean coefficient of detection of 32.96 new cases per 100.000 inhabitants. From all reported cases 140 (7.61%) occurred in children under 15 years old. The age group with the higher proportion of cases (80%; *n* = 112) was between 10 and 14 years of age. The mean coefficient of detection in the population under 15 years was of 7.93 new cases per 100.000 inhabitants. The lowest detection rate in that period was 3.53 cases per 100.000 inhabitants in 2010 and the highest 12.54 cases per 100.000 inhabitants in 2003 (Figure 1).

Table 1 presents the assessment of the degree of deformity at diagnosis and after the cure in leprosy cases reported in children under 15 years.

Degree of deformity at diagnosis was evaluated in 100% of the cases and in 49% (*n* = 69) of the ones discharged after cure.

Table 2 shows clinical and epidemiological features of notified leprosy cases in children under 15 years old, according to their degree of deformity at diagnosis.

The prevalence of physical deformity is higher in males: 13.3% (*n* = 9) presented grade 1 deformity and 8.8% (*n* = 6) grade 2.

Regarding the operational classification, 97.6% of the paucibacillary cases (*n* = 83) showed no physical deformity at diagnosis, and 35.5% of the multibacillary cases (*n* = 19) had some physical deformity.

All indeterminate clinical type cases were classified as grade 0 deformity, and 50% (*n* = 3) of lepromatous cases were diagnosed with physical deformities.

Skin smear positive cases had no hand, feet, or vision impairment at diagnosis. Among skin smear negative cases 30% (*n* = 6) showed grade 1 or grade 2 deformity. It is important to point out that 75,7% (*n* = 106) of the cases did not undergo bacteriological examination or such data was not available.

Among the cases diagnosed by self-referred, 10% (*n* = 8) had grade 1 deformity and 5% (*n* = 4) grade 2. In cases diagnosed by contact examinations, 20% (*n* = 6) showed some physical impairment and 16.7% (*n* = 5) were classified as grade 1 deformity.

In the cases evaluated at discharge, 58 (41.5%) maintained the degree of deformity established at diagnosis; 55 cases (39.4%) had grade 0; and three cases (2.1%) were grade 2 deformity. Physical impairment was increased in three cases: two evolved from grade 0 to grade 1 and one from grade 1 to grade 2. Six cases diagnosed as grade 1 and two cases diagnosed as grade 2 decreased to grade 0.

Table 3 shows that the prevalence of cases with physical deformity was higher in males (PR = 2.65, CI 95%: 1.09 to 6.45), in the multibacillary type (PR = 14.68, CI 95%: 3.54 to 60.87), and lower in passive detection (PR = 0.73, CI 95%: 0.31 to 1.73). For the multivariate analysis were used variables *P* < 0.20 and only one remained statistically significant; therefore, we chose not to interpret the model.

## 4. Discussion

In the analysed period 140 (7.61%) leprosy cases were detected in individuals aged less than 15 years old. The percentage is consistent with the national statistics data which shows that 7% to 8% of notified cases are in such age group [17–19]. Leprosy in children under 15 years old is a public health problem that reflects the transmission cycle of the disease since children may be in a condition of high transmissibility and early exposure to the bacillus. Such factors increase the chances of acquiring and developing the disease [5] and highlight the deficiency of health care services to timely detect cases [19, 20].

The integration of Leprosy Control Activities (LCA) in Brazil started in the 90s with the reorganization of the primary health care through the Community Health Worker Program (in Portuguese PACS) and the Family Health Strategy. In order to obtain this integration, the Brazilian Health Department published ordinances that recommended the implementation of the LCA in the primary health care system [10].

The Unified Health System network must provide care for leprosy patients [14] with the intervention of a multidisciplinary team. Diagnosis is performed by the primary health care physician or at a referral centre on leprosy [21]. The expansion of primary health care services in Brazil contributed to a higher rate of detection of leprosy cases up to 2003. In subsequent years the detection rate of new cases among the general population and the population under 15 years stabilized [8].

Coefficients of detection in the general population and in children under 15 years old are the Department of Health's indicators of the status the endemic situation of leprosy [19]. From 1998 to 2010 the average leprosy detection rates enabled the classification of microregions of Almenara and Araçuaí as very high endemic areas [14]. Other Brazilian municipalities also display high detection rates in children under 15 years old, ranging from very high to hyperendemic levels [5, 19, 22].

All leprosy cases in children under 15 years old had their deformity degree assessed at diagnosis, which is considered good [14]. The incorporation of leprosy control measures in primary health care led to a small increase in patients access to the assessment of their degree of deformity at diagnosis: from 60.9% to 78.1% [18]. Studies carried out in other municipalities in the state revealed coverage of above 90% of the assessment of the degree of deformity at diagnosis [23, 24].

The proportion of new leprosy cases with grade 2 deformity is, as suggested by the WHO, an indicator to monitor disease control actions since it is less susceptible to operational factors such as detection delay when compared with leprosy prevalence [3]. It is worth mentioning that 14.5% of leprosy cases—data not presented—and 5% of cases in children under 15 years old were grade 2 deformity. In the state of Piaui low proportion of physical deformity in children under 15 years old diagnosed between 2003 and 2008 was observed as well [19]. On the other hand, a study carried out in five microregions in the Jequitinonha Valley, in a nine-year time-series analysis (from 1998 to 2006), revealed that 18.6% of the cases presented some kind of deformity at diagnosis [23].

The degree of deformity was more prevalent in males. Similar results were observed among the general population and it may be related to late diagnosis in men [25], women's best access to the health care services [26], and women's greater concern with body image [27].

Regarding the operational classification, studies demonstrate that the vast majority of the leprosy cases diagnosed in children are paucibacillary [5, 22, 28, 29], which is consistent with the observations in Almenara, Araçuaí, and Diamantina. It is remarkable that all cases classified as indeterminate leprosy presented no physical deformities at diagnosis. The indeterminate form of leprosy is expected to be the most common in children due to disease incubation period [5].

Children under 15 years of age diagnosed with multibacillary leprosy showed high prevalence of deformity. Research in the State of Minas Gerais demonstrated that leprosy cases in that age group and in multibacillary leprosy were more likely to show deformities in 7% and 5.7% of the cases, respectively [30].

In most leprosy cases bacilloscopy examination was either not performed or data was not available in the SINAN. The lack of such information points out mistakes when filling out notification forms and indicates the need for instructing health professionals on the importance of registering good quality data. Since dermatological and neurological evaluations are more difficult in children, bacilloscopy and other additional tests are important to confirm diagnosis [28].

Regarding the detection mode, referrals accounted for 18.6% of diagnosis. This may be due to the difficulty of performing dermatological and neurological evaluations in children [22] since responses to skin tests are not reliable [28]. Considering that primary health care professionals may lack experience in identifying leprosy in children, referral centres for diagnosis and followup of difficult cases are essential [28, 31].

It is important to highlight that 20% of leprosy cases diagnosed by contact examination showed some physical impairment when notified. Contact examination is an active mode of detection; therefore delayed diagnosis of the index case or late dermatological and neurological examination of registered contacts might explain such high percentage. Such operational problems may be due to care of leprosy cases being considered as priority of secondary care services. Besides, the fear of social ostracism may dissuade children or their families from seeking medical care at an early stage [20].

A single case, resident in the Almenara microregion, was diagnosed during a group examination. In endemic municipalities it is important to intensify the active case search in order to perform early diagnosis. Most leprosy cases in children under 15 years old were diagnosed by self-referred. In endemic regions, cases diagnosed by self-referred could actually be leprosy contacts of the index case that were not evaluated at the time of notification [27]. The search for contacts is considered an effective method to diagnose the disease as it reduces sources of infection and breaks the chain of transmission. In that age group it is easier to identify index cases which are usually within the family environment [32].

Regarding the assessment of the degree of deformity at discharge, the indicator shows operational precariousness, since 51% of the cases were not evaluated at the end of the treatment [14]. The low assessment percentage of the degree of deformity in leprosy cases at the end of treatment is an operational problem identified in other municipalities too [13, 18, 19, 22, 24]. This situation indicates the prioritization of the assessment of deformity at diagnosis [24], the inadequate followup of patients [19], and failure when feeding the information system [13].

Despite the low number of patients reassessed at discharge, it was possible to observe that most patients maintained the same degree of deformity. Similar results were observed in other studies performed in the region [24]. The low percentage of cases reassessed after cure limits the analysis of this indicator which may be due to failures in control actions or data recording at SINAN.

It is important to emphasize that, at discharge, besides conducting simplified neurological assessment to determine the degree of deformity, health professionals should guide the patients on self-care techniques for the prevention of deformities. One study carried out in Indonesia revealed that persons affected by leprosy face a substantial risk of deteriorating impairments after they are released from treatment [33].

Furthermore, it is important to monitor the degree of deformity after discharge with the aim of preventing nerve damage [25], activity limitations, stigma, discrimination, and social restriction [33]. Monitoring of physical deformity after treatment is a challenge to the public health system. It is necessary to invest in self-care education and physical rehabilitation [33].

## 5. Final Considerations

Prevalence of physical deformities was higher in males and in multibacillary leprosy cases. There was high detection rate of leprosy in the general population and in patients under 15 years old and high proportion of grade 2 deformity. Active search measures as well as group and household contact examinations were precarious. Performance of bacteriological examination and assessment of the degree of deformity at discharge were inadequately covered.

The studied context suggests high transmissibility and early exposure to *Mycobacterium leprae* since a lot of cases were diagnosed in children under fifteen years old and the incubation period of the leprosy bacillus varies from 02 to 07 years. Such situation contributes to maintenance chain of disease transmission in the region. Efforts to control the disease should be increased. More investment in health professionals training and educational activities regarding the signs and symptoms of the disease is needed.

Furthermore, it is important to intensify the search for new cases and household contacts. Early diagnosis might break the chain of transmission and reduce the physical, psychological, social, and behavioural burden of the disease.

## Figures and Tables

**Table 1:** Degree of deformity evaluation of notified leprosy cases in children under 15 years old in Almenara, Araçuaí, and Diamantina between 1998 and 2010.

Evaluation of deformity	*n*	%
Diagnostic		
Degree 0	119	85,0
Degree 1	14	10,0
Degree 2	7	5,0

Total	140	100,0

Discharged		
Degree 0	63	91,3
Degree 1	2	2,9
Degree 2	4	5,8

Total	69	100,0

**Table 2:** Clinical and epidemiological features of notified leprosy cases in children under 15 years old according to their degree of deformity in Almenara, Araçuaí, and Diamantina between 1998 and 2010.

	Degree of physical deformity		
Variable	0	1	2
	*n* (%)	*n* (%)	*n* (%)
Gender			
Female	66 (91,7)	5 (6,9)	1 (1,4)
Male	53 (77,9)	9 (13,3)	6 (8,8)
Operational classification*			
Paucibacillary	83 (97,6)	2 (2,4)	—
Multibacillary	36 (65,5)	12 (21,8)	7 (12,7)
Clinical forms**			
Indeterminate	66 (100)	—	—
Tuberculoid	17 (89,5)	2 (10,5)	—
Borderline	33 (67,3)	10 (24,4)	6 (12,2)
Lepromatous	3 (50,0)	2 (33,3)	1 (16,7)
Skin smear***			
Negative	24 (80,0)	3 (10,0)	3 (10,0)
Positive	4 (100,0)	—	—
Not performed	17 (100,0)	—	—
Ignored	74 (75,7)	11 (12,8)	4 (4,7)
Detection mode****			
Referral	23 (88,5)	1 (3,8)	2 (7,7)
Self-referred	68 (85,0)	8 (10,0)	4 (5,0)
Group examination	1 (100,0)	—	—
Contact examination	24 (80,0)	5 (16,7)	1 (3,3)
Ignored	3 (100,0)	—	—

Total	119 (85,0)	14 (10,0)	7 (5,0)

*World Health Organization classification according to number of skin lesions and nerve involvement: paucibacillary (up to 05 lesions and involvement of only one nerve) and multibacillary (more than 05 lesions and involvement of more than one nerve).

**Clinical form based on the Madrid classification.

***Not performed: skin smear was not performed; ignored: the information is not available at SINAN.

****Referral: at referral centre; self-referred: patients report on their own to health centre; group examination: mass surveys and campaigns; contact examination: detection by examination of household contacts.

**Table 3:** Prevalence ratio and confidence intervals of factors associated with physical deformity of notified leprosy cases in children under 15 years old in Almenara, Araçuaí, and Diamantina between 1998 and 2010.

	Degree of physical deformity at diagnosis						
Variable	Yes		No		PR	*P* value	CI 95%
	*n*	%	*n*	%			
Gender							
Female	6	8,3	66	91,7	1		
Male	15	22,1	53	77,9	2,65	0,032	1,09–6,45
Operational classification*							
Paucibacillary	2	2,4	83	97,6	1		
Multibacillary	19	34,5	36	65,5	14,68	<0,001	3,54–60,87
Detection mode							
Active	6	19,4	25	80,6	1		
Passive	15	14,2	91	85,8	0,73	0,476	0,31–1,73

PR: prevalence ratio; *P* value: Poisson regression; CI: confidence intervals.

*World Health Organization classification according to number of skin lesions: paucibacillary (up to 05 lesions) and multibacillary (more than 05 lesions).
